# Gastric bypass surgery has a weight-loss independent effect on post-challenge serum glucose levels

**DOI:** 10.1186/s13098-015-0066-8

**Published:** 2015-08-22

**Authors:** Dag Hofsø, Kåre I. Birkeland, Jens J. Holst, Jens Bollerslev, Rune Sandbu, Jo Røislien, Jøran Hjelmesæth

**Affiliations:** Morbid Obesity Centre, Vestfold Hospital Trust, post box 2168, 3103 Tønsberg, Norway; Department of Endocrinology, Morbid Obesity and Preventive Medicine, Oslo University Hospital, Oslo, Norway; Faculty of Medicine, University of Oslo, Oslo, Norway; Department of Biomedical Sciences, Endocrinology Research Section, Copenhagen, Denmark; Section of Endocrinology, Department of Medicine, Oslo University Hospital Rikshospitalet, Oslo, Norway; Department of Surgery, Vestfold Hospital Trust, Tønsberg, Norway; Department of Biostatistics, Institute of Basic Medical Sciences, University of Oslo, Oslo, Norway

**Keywords:** Morbid obesity, Hypoglycaemia, Gastric bypass surgery

## Abstract

**Background:**

Gastric bypass surgery seems to have an effect on glucose metabolism beyond what is mediated through weight reduction. The magnitude of this effect on fasting and post-challenge glucose levels remains unknown.

**Results:**

Morbidly obese subjects without known diabetes performed a 75 g oral glucose tolerance test before and after either gastric bypass surgery (n = 64) or an intensive lifestyle intervention programme (n = 55), ClinicalTrials.gov identifier NCT00273104. The age-adjusted effects of the therapeutic procedures and percentage weight change on fasting and 2-h glucose levels at 1 year were explored using multiple linear regression analysis. Mean (SD) serum fasting and 2-h glucose levels at baseline did not differ between the surgery and lifestyle groups. Weight-loss after surgical treatment and lifestyle intervention was 30 (8) and 9 (10) % (p < 0.001). At 1 year, fasting and 2-h glucose levels were significantly lower in the surgery group than in the lifestyle group, 4.7 (0.4) versus 5.4 (0.7) mmol/l and 3.4 (0.8) versus 6.0 (2.4) mmol/l, respectively (both p < 0.001). Gastric bypass and weight-loss had both independent glucose-lowering effects on 2-h glucose levels [B (95 % CI) 1.4 (0.6–2.3) mmol/l and 0.4 (0.1–0.7) mmol/l per 10 % weight-loss, respectively]. Fasting glucose levels were determined by weight change [0.2 (0.1–0.3) mmol/l per 10 % weight-loss] and not by type of treatment.

**Conclusions:**

Gastric bypass surgery has a clinically relevant glucose-lowering effect on post-challenge glucose levels which is seemingly not mediated through weight-loss alone.

## Background

Reduction in blood glucose levels after gastric bypass surgery is related to weight-loss [1, 2]. In addition, a weight-loss independent effect of the surgical procedure on glucose metabolism has been hypothesized [2, 3]. Exaggerated post-prandial insulin secretion observed after gastric bypass supports this hypothesis [4–7]. The relative effects of weight loss and the surgical procedure per se on fasting and post-challenge glucose levels remain, however, unknown.

The aim of the present analysis was to assess the effects of weight-loss achieved by gastric bypass surgery and lifestyle intervention on 1 year fasting and 2-h glucose levels. Data from oral glucose tolerance tests (OGTTs) in the previously published “Morbid Obesity treatment, Bariatric surgery versus Intensive Lifestyle intervention” (MOBIL) study [1] were analysed. The inclusion of a non-surgical weight-loss group made it possible to assess the specific effect of the surgical procedure on fasting and post-challenge glucose levels. To our knowledge, this has previously only been addressed in a few small studies [8, 9].

## Methods

This is a post hoc analysis of data from the MOBIL study [1] conducted at a public tertiary care centre in Norway. The trial included 146 morbidly obese subjects of mainly European descent, and aimed to address changes in several health outcomes related to obesity after gastric bypass surgery or an intensive lifestyle intervention programme. A 75 g 2-h OGTT was performed in subjects without known diabetes (119 out of 139 completers) at the time of inclusion. The present analyses include data from these participants. Some of the previously published data are included in the present paper as they are essential for interpretative purposes [1, 7].

Patients in the surgery group received laparoscopic Roux-en-Y gastric bypass, whereas patients who chose lifestyle intervention were referred to a rehabilitation centre specialising in the care of morbidly obese patients (Evjeklinikken A/S, Evje, Norway). The 1 year lifestyle programme at the rehabilitation centre aimed to induce a weight-loss of at least 10 % and comprised of four stays at the centre lasting for either 1 or 4 weeks (total 7 week stay).

The regional ethics committee of the Southern Norway Regional Health Authority approved the study. The study is registered in the ClinicalTrials.gov-registry under the unique trial number NCT00273104. Written informed consent was provided by all participants.

Data are presented as mean (SD) or number (%). Unadjusted between-group comparisons were assessed using either independent samples *t* test or the Chi square test. Multiple linear regression analyses were used to assess the associations between treatment choice and weight change on serum glucose levels at 1 year. Due to age differences between the groups at baseline and the known impact of age on both fasting and 2-h glucose levels, the effects of the independent variables were age-adjusted. Interaction terms between the two explanatory variables (group and weight change) were included. Due to a standardised residual of 4.81 one case was excluded from the correlation and regression analyses including 2-h glucose as the dependent variable. The significance level was p < 0.05. Statistical analyses were performed using SPSS 17.0 (SPSS Inc., Chicago, IL, USA).

## Results

Patients in the surgery group were younger [41 (10) versus 46 (11) years, p = 0.007], heavier [47 (6) versus 43 (5) kg/m^2^, p < 0.001] and had greater weight-loss at one-year follow-up [30 (8) versus 9 (10) %, p < 0.001]. The proportion of females did not differ between the surgery and lifestyle groups (70 versus 71 %, p = 0.943). Fasting and 2-h glucose levels at baseline did not differ between the surgery and lifestyle groups, 6.1 (1.6) versus 6.0 (1.0) mmol/l, p = 0.495 and 7.5 (3.4) versus 7.6 (3.1) mmol/l, p = 0.887. In contrast, both fasting and 2-h glucose levels were significantly lower in the surgery group than in the lifestyle group at 1 year, 4.7 (0.4) versus 5.4 (0.7) mmol/l and 3.4 (0.8) versus 6.0 (2.4) mmol/l (both p < 0.001).

**Fig. 1:**
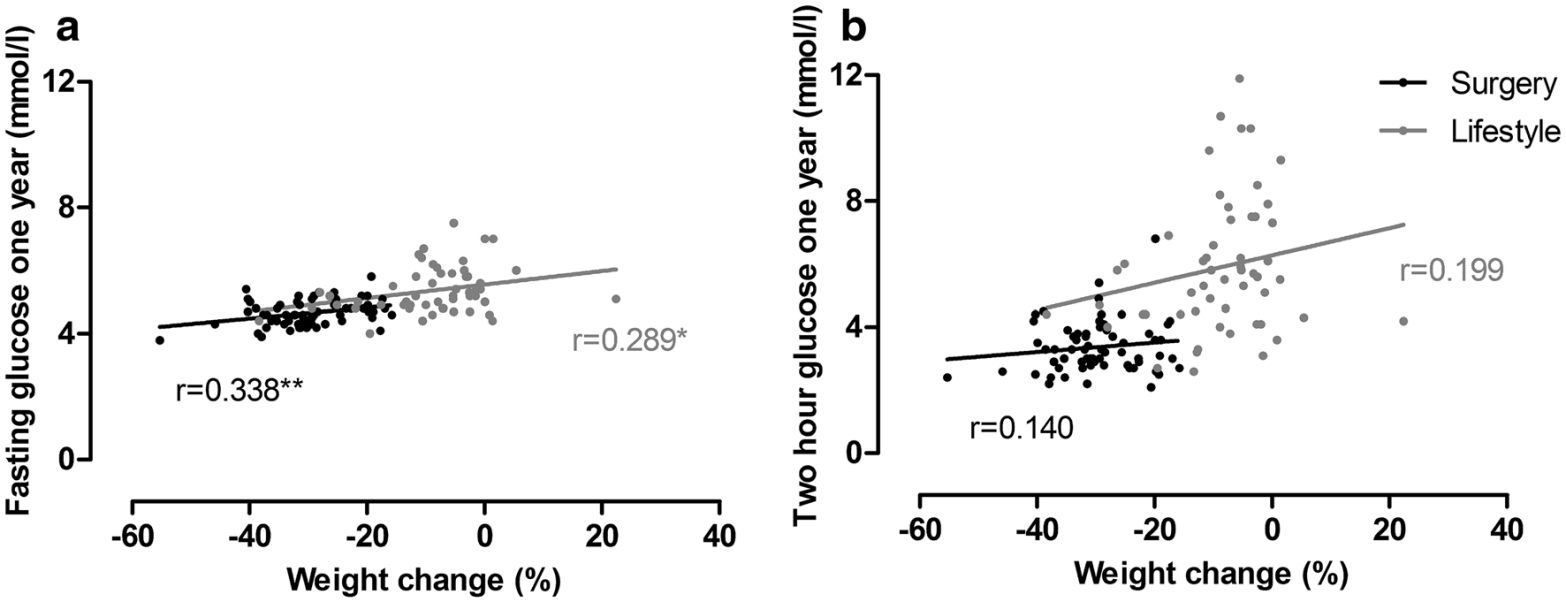
Correlations between percentage weight change and fasting and 2-h glucose levels at 1 year in the surgery and lifestyle groups. **p < 0.01, *p < 0.05

The correlations between percentage weight change and fasting and 2-h glucose levels 1 year after surgical and medical treatment are shown in Fig. 1. Weight change correlated significantly with fasting glucose levels at 1 year in both groups. Further, the independent effects of treatment choice (group) and weight change on fasting and 2-h glucose levels were explored in two regression models (Table 1). Gastric bypass surgery had an independent effect on 2-h glucose levels but not on fasting glucose levels at 1 year (Table 1). Nine percent of the variance in 2-h glucose was explained by treatment choice. The mean (95 % CI) glucose-lowering effect of surgical treatment on 2-h glucose was 1.4 (0.6–2.3) mmol/l. The distance between the group-specific correlation lines in Fig. 1b visualises this effect. Weight change per se was independently associated with both fasting and 2-h glucose levels and explained 13 and 5 % (squared partial correlation) of the variance in the two glucose variables, respectively (Table 1). A 10 % weight loss was associated with a reduction in fasting glucose of approximately 0.2 mmol/l and a reduction in 2-h glucose of approximately 0.4 mmol/l (B × 10, Table 1). The associations between weight change and fasting and 2-h glucose levels in the two groups did not differ significantly (no “group × weight change” interactions, p = 0.371 and p = 0.130, respectively). Age was positively associated with both fasting glucose levels and 2-h glucose levels and explained 18 and 13 % of the variance in the respective glucose variables (Table 1). Overall, treatment choice, weight change and age explained 46 % (R^2^ = 0.459) and 49 % (R^2^ = 0.491) of the variance in fasting and 2-h glucose levels 1 year after treatment.

**Table 1:** Multiple linear regression analyses with treatment choice, weight change and age as explanatory variables and fasting and 2-h serum glucose levels at 1 year as outcome variables

	Fasting serum glucose			2-h serum glucose		
	B (95 % CI)	Squared partial correlation	P value	B (95 % CI)	Squared partial correlation	P value
Group	0.118 (−0.177 to 0.414)	0.005	0.429	1.443 (0.585–2.301)	0.089	0.001
Weight change (%)	0.022 (0.012–0.033)	0.132	<0.001	0.038 (0.007–0.068)	0.049	0.017
Age (years)	0.022 (0.013–0.031)	0.177	<0.001	0.054 (0.028–0.079)	0.132	<0.001

Group: surgery = 0, lifestyle = 1

## Discussion

The main finding of the present study is that gastric bypass surgery seems to have an effect on post-challenge glucose levels that is partly independent of weight loss, while the effect on fasting glucose levels is solely dependent on the degree of weight loss.

These results support the idea that gastric bypass surgery has a direct effect on glucose metabolism beyond what is mediated by weight change [3]. However, the effect seems to be specific for the post-prandial phase. In line with previous studies the procedure does not seem to have an independent effect on fasting glucose levels [8, 9]. Improved fasting glucose is likely to be caused by reduced endogenous hepatic glucose production associated with weight-loss [10]. Contrasting our findings, changes in post-prandial glucose did not differ significantly after 10 % weight-loss induced by either gastric bypass or lifestyle intervention [9]. However, the study seems inadequately powered (n = 20) for detecting clinically relevant differences in post-prandial glucose levels. Supra-physiological post-prandial insulin secretion [4–7], possibly due to alteration of the gastrointestinal tract [11] with rapid glucose absorption [4] and exaggerated incretin response [4–6], may possibly explain the weight-loss independent effect of gastric bypass on 2-h glucose levels observed in the present study.

The weight-loss independent glucose lowering effect of gastric bypass surgery on 2-h glucose levels might be beneficial in subjects with post-prandial hyperglycaemia. However, the potent effect of gastric bypass on post-challenge glycaemia may also increase the risk of post-prandial hypoglycaemia. This side effect is increasingly recognised to be a potential result of gastric bypass surgery [12]. Age and weight change were both significantly and positively associated with 2-h glucose. These findings may suggest that younger subjects who experience large weight reduction after surgery may have an increased risk of developing post-prandial hypoglycaemia.

Most of the patients included in this analysis had glucose levels within the normal or pre-diabetic range, meaning that the results cannot be generalised to include patients with overt diabetes. Due to the non-randomised design of the study we cannot exclude that between-group differences may have confounded our interpretation of the data. However, no differences in glucose levels at baseline were observed. Moreover, statistical adjustments for differences in age between the two groups were performed in order to minimise any confounding.

## Conclusions

The strong post-challenge glucose lowering effect of gastric bypass surgery on 2-h glucose is partly independent of weight-loss. This finding supports the presence of gastric bypass specific glucose regulatory mechanisms.

## Abbreviations

OGTT: oral glucose tolerance tests
MOBIL: morbid obesity treatment, bariatric surgery versus intensive lifestyle intervention
